# Weight management experiences and needs in obese polycystic ovary syndrome patients of childbearing age in China: a qualitative study

**DOI:** 10.1590/1980-220X-REEUSP-2024-0354en

**Published:** 2025-04-14

**Authors:** Hong Jiang, Min Luo, Hongjin Wu, Xixi Li

**Affiliations:** 1 Chengdu Medical College, School of Nursing, Chengdu, Sichuan, China.; 2 University of Electronic Science and Technology of China, School of Medicine, Nursing Department, Mianyang Central Hospital, Mianyang, Sichuan, China.; 3 903 Hospital, Chengdu Medical College, Jiangyou, Mianyang, Sichuan, China.

**Keywords:** Polycystic Ovary Syndrome, Reproductive Health, Obesity, Síndrome do Ovário Policístico, Obesidade

## Abstract

**Objective:**

To explore the weight management experiences and needs of obese polycystic ovary syndrome patients of reproductive age in China.

**Method:**

Thirteen patients were recruited from a tertiary hospital in Sichuan province. Face-to-face structured interviews were conducted using a phenomenological approach, with data analyzed via Colaizzi seven-step method.

**Results:**

Key weight management methods identified included medication, diet, exercise, and habit correction. Coping attitudes varied, with some patients exhibiting positive or negative responses. Experiences revealed short-term adherence to dietary control but difficulty maintaining exercise routines. Patients expressed a need for support from medical staff and family, as well as interest in medications and traditional Chinese medicine to aid weight loss.

**Conclusion:**

Insights into the weight management experiences and needs of obese PCOS patients can guide medical staff in addressing these challenges. Attention to patients' and families' emotional well-being during weight management is also crucial for effective intervention.

## RESUMO

**Objetivo:**

Explorar as experiências e necessidades de controle de peso de pacientes obesas com síndrome dos ovários policísticos em idade reprodutiva na China.

**Métodos:**

Treze pacientes foram recrutadas em um hospital terciário na província de Sichuan. Foram realizadas entrevistas estruturadas presenciais com abordagem fenomenológica, com dados analisados pelo método de sete etapas de Colaizzi.

**Resultados:**

Os principais métodos de controle de peso identificados incluíram medicamentos, dieta, exercícios e correção de hábitos. As atitudes de enfrentamento variaram, com alguns pacientes apresentando respostas positivas ou negativas. As experiências revelaram adesão de curto prazo ao controle alimentar, mas dificuldade em manter rotinas de exercícios. As pacientes expressaram necessidade de apoio da equipe médica e da família, bem como interesse em medicamentos e medicina tradicional chinesa para auxiliar na perda de peso.

**Conclusão:**

Insights sobre as experiências e necessidades de controle de peso de pacientes obesas com SOP podem orientar a equipe médica na abordagem desses desafios. A atenção ao bem-estar emocional dos pacientes e familiares durante o controle do peso também é crucial para uma intervenção eficaz.

## INTRODUCTION

The World Health Organization (WHO) defines women of reproductive age as those aged 15–49 years^(1)^. This demographic is critical, as their health impacts extend beyond thier individual well-being to those of families and communities. The WHO emphasizes that addressing female reproductive health is essential for improving maternal and child health outcomes and achieving broader societal and economic goals^(2)^. The Programme for the Development of Chinese Women (2021–2030)^(3)^ prioritizes “women and health” to improve reproductive health and address challenges faced by women of reproductive age. The central focus of this program is tackling critical health issues such as infertility, a critical issue with a prevalence of 5.8% (2006–2010) to 8.1% (2017–2019), affecting approximately 10% to 15% of couples^(4)^. Polycystic ovary syndrome (PCOS) is the leading cause of anovulatory infertility in up to 15% of women worldwide and affects women of reproductive age with a global prevalence of 6-20%^(5)^.PCOS is characterized by menstrual irregularities, hyperandrogenism (manifesting as hirsutism and acne), and polycystic ovaries on ultrasound imaging^(6)^. In addition to reproductive health issues, PCOS-related insulin resistance increases the risk of type 2 diabetes (up to 28% by age 40), as well as hypertension, high cholesterol, and atherosclerosis, all of which elevate the risk of heart disease and stroke^(7)^. These complications not only reduce quality of life but also strain healthcare systems, with long-term care and medication increasing costs^(8)^. Patients with PCOS are predisposed to obesity, which can exacerbate their condition^(9)^. The interaction between PCOS and obesity often creates a vicious cycle, further complicating the health outcomes of affected individuals, and symptoms. However, weight loss of 5–10% of body weight has been shown to improve PCOS symptoms^(10)^. Effective weight management is essential in patients with obesity with PCOS. Although numerous weight management strategies are available for these patients, their adherence is generally low^(11)^.

Therefore,to further understand the experiences and weight management needs of obese women with PCOS, this study employs qualitative methods, in-depth interviews with this patient group. The insights gathered will provide recommendations for patient-centered care strategies, ensuring that the voices of women living with PCOS are central to improving healthcare outcomes and informing future policies.

## METHOD

### Design and Setting

This study used a qualitative method of semi-structured interviews to find out the experiences and practical needs of Chinese obese patients with PCOS of reproductive age. The method of qualitative inductive content analysis was used to describe weight management experiences and needs in obese polycystic ovary syndrome patients of reproductive age in China.Results were presented according to the consolidated criteria for reporting qualitative research (CO-REQ).

### Methods

This study employed a qualitative design to align with its objective of exploring patient experiences. Semi-structured interviews were conducted with women diagnosed with PCOS, focusing on their challenges, needs, and strategies related to weight management.

### Population, Inclusion and Exclusion Criteria

Using the purposive sampling method, obese PCOS patients who were enrolled in the gynaecological and endocrinology outpatient clinic of one tertiary general hospital in Sichuan province in China, between March 2023 to April 2023. Patients were eligible for inclusion based on these criteria: 1) patients of reproductive age diagnosed with PCOS^(12)^; 2) Body Mass Index (BMI) ≥ 30kg/m^2(13)^; 3) normal mental consciousness and good communication ability. We excluded patients who had: 1) patients who automatic withdrawal during the interview; 2) patients who could not provide contact details; 3)patients who have been pregnant.

### Interview and Procedure

Through the literature review, the research team initially designed the interview outline, conducted pre-interviews with three patients who met the criteria, revised the interview outline according to the interview, and finally determined the formal interview outline. The modified interview guide questions for each participant were listed in Table 1.

**Table 1:** Interview Guide – Mianyang, Sichuang, China, 2023.

Number	Questions
1	Do you know something of PCOS?
2	Do you currently manage your weight? If so, how do you control your weight?
3	Do you have any feelings when managing your weight?
4	What is your motivation for weight management?
5	Do you find any difficulties in your weight management process?
6	What is the effectiveness of your current weight control program?
7	Is there anything you need help with regarding weight management?

This study employed one-on-one semi-structured interviews using a phenomenological approach. Prior to the interviews, the researcher communicated the study’s purpose and methodology to the respondents, who then provided informed consent. All interviews were conducted in a quiet, private outpatient filing room and lasted approximately 30 minutes. Before the interview, the interviewees were assured that their participation would not affect the quality of their future treatment. A voice recorder was used to record the interviews, and a trained researcher following a pre-established interview guide. The researcher remained neutral, refraining from leading questions or biased evaluations, and avoided interrupting the participants. During the interview, the researcher paied attention to the subjects’ facial expressions and body movements during the interview, and timely recorded.

### Data Analysis

After each interview, the audio recordings were transcribed into textual material within 24 hours by two trained professionals working independently. NVIVO 12 software was used to organize and manage the textual data. The analysis followed Colaizzi seven-step analysis method^(14)^ to process the data systematically. The first author revisited the original interview data to identify preliminary themes. These themes were then compared with those derived independently by the two trained professionals. Discrepancies were discussed and resolved collaboratively, leading to a set of proposed themes. Finally, all members of the research team reviewed the proposed themes and reached a consensus to establish the final themes.

### Ethical Approval

All methods were performed in accordance with the relevant guidelines and regulations or in accordance with the Declaration of Helsinki. The Ethics Committee approval of the Mianyang Central Hospital, School of Medicine, University of Electronic Science and Technology of China before conducting the study (ID: S20230306-02). This study was approved by the Ethics and Clinical Investigation Committee of Hospital, with exemption granted on the need for informed consent.

## RESULTS

### Demographic Characteristics of Participants

The sample size of this study was based on information saturation, and a total of 13 obese PCOS patients were finally included. In this study, according to the principle of confidentiality, D1–D13 were used in this study to represent the interviewees, and the general information of the interviewees is shown in Table 2.

**Table 2:** General information about the interviewees (n = 13) – Mianyang, Sichuang, China, 2023.

Number	Age (year)	Educational background	Marital and reproductive status	Occupation	Currently reproductive need
D1	30	Senior high school graduate	Married with children	Unemployed	No
D2	35	Senior high school graduate	Married with children	Non-government employee	Yes
D3	24	Junior high school graduate	Married with no children	Non-government employee	Yes
D4	18	Senior high school student	Unmarried and childless	Student	No
D5	20	College student	Unmarried and childless	Student	No
D6	26	Senior high school graduate	Married with no children	Non-government employee	Yes
D7	25	College graduate	Married with no children	Unemployed	Yes
D8	28	College graduate	Married with children	Merchant	No
D9	20	College student	Unmarried and childless	Student	No
D10	27	Senior high school graduate	Unmarried and childless	Merchant	Yes
D11	23	College graduate	Unmarried and childless	Government employee	No
D12	20	College student	Unmarried and childless	Student	No
D13	23	Master candidate	Unmarried and childless	Student	No

Through detailed analysis of the transcribed interviews, four themes were identified: the methods of weight management in obese PCOS patients, obese PCOS patients’ attitudes to weight management coping, obese PCOS patients’ experiences of weight management practices, and obese PCOS patients’ weight management needs, as shown in Table 3.

**Table 3:** Key Themes and Sub-Themes – Mianyang, Sichuang, China, 2023.

Themes	Sub-Themes
The methods of weight management in obese PCOS patients	1. Medication management
	2. Diet management
	3. Exercise management
	4. Correction of unreasonable habits
The coping attitude of weight management in obese PCOS patients	1. Positive coping attitude
	2. Negative coping attitude
The experiences of weight management methods in obese PCOS patients	1. Easy to adhere to dietary control in a short term
	2. Difficult to adhere to exercise
The weight management needs in obese PCOS patients	3. Need help for medical staff
	4. Need help for their families
	1. Wish to use medication to assist in weight reduction
	2. Wish to use traditional Chinese medicine to assist in weight reduction

### Theme 1: The Methods of Weight Management in Obese Pcos Patients

#### Sub-Theme 1: Medication Management

Some interviewees had a combination of insulin resistance or type 2 diabetes mellitus, and their doctors prescribed medications such as metformin and simethicone to manage the condition, which also helped with weight control. Most patients reported positive effects on weight management, though a small group exhibited poor adherence to the prescribed medications. Additionally, some patients had purchased weight-loss drugs through unofficial channels, but this group experienced weight gain after discontinuing the use of these drugs.

*I purposely went to a Chinese medicine practitioner, who prescribed me some oral and topical Chinese medicines. (D2)*

*Before I was actually only 72kg, I felt very fat at that time and bought weight-loss drugs (on the Internet) to eat, as a result, I have now grown to 85kg. (D10)*

*The doctor prescribed me Metformin, and Semaglutide injection, she said that these medicines help me to control my blood sugar and lose weight, so far it’s very effective, I’ve lost 7.5kg. (D11)*

*My doctor prescribed me metformin, but I often forget to take my medication. (D13)*

#### Sub-Theme 2: Diet Management

All interview subjects in this study underwent dietary changes (including adjustments to food structure, calorie restrictions, and changes in eating times and so on) after being diagnosed with PCOS. However, they still lacked professional nutrition knowledge.

*I used to be particularly fond of foods like rice and noodles. Since I was checked for PCOS, I’m now eating one small bowl of rice because they (close friends) said it’s not good to consume too many starchy foods. (D1)*

*Now, I just drink soya milk and eat eggs and whole meal bread in the morning, chicken breast and some rice and lots of vegetables for lunch, and basically cucumber, eggplant, tomatoes and lettuce for dinner. (D2)*

*After I was checked out by the doctor, I eat lighter. (D4)*

*Compared to before, I eat fewer total calories now, but I’m still not sure exactly how to take it. (D7)*

#### Sub-Theme 3: Exercise Management

Only one interviewee in this study set an exercise programme for herself and followed it regularly daily. In contrast, the rest of the interviewees exercised little or none.

*I don’t exercise very deliberately,because I’m busy most of the time. (D1)*

*I exercise every day. I give myself a rule to walk more than 6,000 steps and jump rope more than 100 times every day. (D2)*

*Occasionally I exercise, but overall I don’t spend much time exercising. (D9)*

*I didn’t used to exercise, after I found out about this disease (PCOS), I started to do gymnastics, but only when I’m not at work, so it’s just about 3-4 times a month that I can do gymnastics for about half an hour each time. (D11)*

#### Sub-Theme 4: Correction of Unreasonable Habits

Most respondents in this study subjectively believed they had improved problematic habits, such as drinking more water, snacking less, limiting alcohol, and sleeping earlier. However, while many recognized and improved some of their unhealthy habits, they did not correct all of them.

*Now I eat very few crisps and haven very little milk tea, anyway, have a snack once in a while. (D5)*

*I’m in business and often eat in restaurants. But now I drink (baijiu) less because I have to talk business with others and it’s impossible not to drink. (D10)*

*I don’t smoke anymore, sometimes I still drink some beer, I used to go to bed after 3am but now I go to bed before 1am. (D13)*

### Theme 2: Coping Attitude of Weight Management in Obese Pcos Patients

#### Sub-Theme 1: Positive Coping Attitude

Many respondents indicated that weight control is beneficial for disease recovery and emphasized the importance of cooperating with their doctors. They also believed that maintaining a positive mindset is crucial for long-term weight loss success. Additionally, seeing the improved appearance of other patients after successful weight loss motivated them to continue their efforts.

*Controlling weight is a difficult thing. Anyway, it is a long battle, but I am very confident. This is because people still need positive examples, and I want to set an example for my children. (D1)*

*The most significant thing to me is my health, and I understand what I need to do to keep my weight under control. If I control it well I won’t get diabetes, endometrial cancer and all that. (D2)*

*I just want to be thin and look beautiful. Before there was a patient online who also had this disease. She lost weight and became very attractive. (D4)*

#### Sub-Theme 2: Negative Coping Attitude

 All respondents in this study were patients with chronic obesity. Some had lost confidence in their ability to lose weight due to their long struggle with obesity and previous failures, and no longer believed successful weight loss was possible for them.Thus these patients were reluctant to engage in weight management.

*No matter what I do, I am unable to lose weight for many years.(D10)*

*In fact, I feel that I eat about the same as the normal weight people around me, but I am just fatter than others, so I feel that it’s unfair. So I don’t think there’s any need to lose weight, so that’s it. (D11)*

*I’m just a very common big fat person, I’ve been fat since I was a kid, I just like to eat and sleep, I don’t want to exercise, it doesn’t matter anyway, I’m not getting married or having kids. (D12)*

### Theme 3: The Experiences of Weight Management Methods in Obese Pcos Patients

#### Sub-Theme 1: Easy to Adhere to Dietary Control in a Short Term

Most of the patients said that they were very willing to lose weight through dietary control and could adhere to strict diets in a short term. But they would still like a dietary plan in the long term, and that supervision by someone else would be better to help them adhere to their diets.

*I’d still like to be able to snack a couple of times a month if possible, otherwise I will always crave those snacks. (D4)*

*Now, I don’t eat any food at night. (D10)*

*I used to have milk tea and snacks, but now I don’t eat these anymore. I’ve been sticking to it for two months, but I definitely can’t do it in the long run without snacking. (D11)*

*At school my friends will supervise me, at home my mother will supervise me. (D12)*

#### Sub-Theme 2: Difficult to Adhere to Exercise

Most patients have difficulty adhering to exercise. Office workers often avoid exercise due to the physical and mental strain of their jobs, while students struggle to find time because of academic demands. Some patients discontinue exercise for physical, psychological, or urgent reasons.

*I am too tired after work , so I don’t want to exercise. (D3)*

*I’m in my third year of high school now, soI have to spend a lot of time studying, it’s impossible for me to exercise every day. (D4)*

*There are a lot of things in real life that can disrupt your plans, like suddenly there’s something going on at home or something like that, and it causes me not to keep exercising. (D8)*

*I hurt my knee by exercising before, and now I don’t dare to exercise on my own. (D9)*

### Theme 4: The Weight Management Needs in Obese Pcos Patients

#### Sub-Theme 1: Need Help for Medical Staff

All patients expressed their wish to receive support from the medical staff, to gain more knowledge about the disease, to know the impact of obesity on the disease, and to build up confidence in weight reduction and succeed.

*I have been diagnosed with PCOS for 7 years. To be honest, I can lose my weight very quickly but then bounce back later on. I still think it is not good for my health, I should have to come to see the doctor in the hospital regularly. (D2)*

*I don’t know specifically what the consequences of my illness are, I just know that if I can’t lose the weight now I won’t be able to get pregnant easily. I’d really like to lose the weight quickly, so I’d just like to ask the doctors and nurses in the hospital more questions about what I should be doing right now. (D3)*

*Anyway, I’ve used all the ways I can lose weight on my own, and now I really can’t do anything else, so that’s why I came to the hospital to lose weight. (D12)*

#### Sub-Theme 2: Need Help for Their Families

Inadequate family support for some patients leads to impediments to weight loss and abnormal emotions due to lack of care from family.

*When I was losing weight, my family would purposely lure me in with some delicious food. I would just like my family members not to do that when I am losing weight. (D5)*

*I feel like my family members only cares about when I can become pregnant, and I’ve told them that I need to reduce my weight before I can have a baby more easily. However, they would only push me to get pregnant faster. (D7)*

*I take care of my child every day, I don’t have time to exercise, before I wanted to go to a closed camp to lose weight, but when I went there no one took care of my child. (D8)*

#### Sub-Theme 3: Wish to Use Medication to Assist in Weight Reduction

Some patients expressed a desire to be prescribed medication to lose weight. They wanted to lose weight by controlling their appetite and increasing their metabolism.

*I just need you guys to prescribe me diet pills now, I can’t wait to lose weight, I want to get pregnant soon. (D6)*

*I want to lose weight quickly now, it’s better to prescribe me some diet pills, I can never lose weight on my own. (D10)*

*I hope you will prescribe me some medicine that can control my appetite. Before I had a very strict diet plan, but sometimes I eat much food in and regret it, then I go to vomit. (D11)*

#### Sub-Theme 4: Wish to use Traditional Chinese Medicine to Assist in Weight Reduction

Most patients expressed a desire for help from traditional Chinese medicine practitioners, believing that treatments like acupuncture, acupoint therapy, and buried wires could aid in weight loss. Additionally, they thought soaking their feet in specially formulated Chinese herbal medicines could help promote metabolism.

*Many people say that traditional Chinese medicine can regulate menstruation and help you lose weight. (D1)*

*Traditional Chinese medicine is really useful. I have a friend who also has PCOS, she has prescribed some Chinese medicines from an Chinese doctor, now not only is her menstruation normal, but she has also become younger and prettier. And most of all, her weight has gone down. (D2)*

*I recently had acupuncture at a traditional Chinese medicine centre outside, they said that acupuncture can help me lose weight, and at the moment I did lose my weight as well. (D12)*

## DISCUSSION

Studies have reported that women with PCOS experience varying degrees of anxiety (37%) and depression (42%), and factors such as obesity and previous failed weight loss experiences significantly increase their psychological burden^(15, 16)^. All interviewees in this study were obese PCOS patients, who had previously attempted failed weight loss programs and experienced weight regain. Anxiety and depression among patients with PCOS often manifest in various ways, including persistent feelings of sadness, hopelessness, and worthlessness. These patients may also experience symptoms such as sleep disturbances, difficulty concentrating, and excessive worry about their health and future. Another study highlighted that severe psychological distress was associated with an increased risk of self-harm and reduced quality of life^(17)^. These mental health challenges can also exacerbate endocrine dysfunction, lower conception rates, and negatively affect overall quality of life^(18)^. Therefore, it is critical to systematically assess and intervene as early as possible when patients have symptoms of anxiety and depression to reduce disease burden and improve prognosis.

However, studies have revealed that only a small percentage of doctors in China actively address the psychological problems of patients with PCOS^(19)^. This gap may be attributed to its large population base, significant number of patients, and insufficient medical resources, which results in doctors paying attention only to patients’ physical illnesses and neglecting their psychological conditions. It is impossible to conduct in-depth interviews of all patients with obesity and PCOS to determine their psychological status. It is recommended that all such patients undergo routine psychological screening using standardized tools such as the Generalized Anxiety Disorder Scale or Patient Health Questionnaire^(20)^. Repeat screenings should be conducted based on clinical judgment and disease progression, particularly at critical times (e.g., when patients exhibit mood changes or reach significant treatment milestones). Timely targeted interventions are essential for patients with abnormal emotional states. Evidence-based therapeutic approaches, including cognitive-behavioral therapy and lifestyle interventions such as structured exercise and dietary modifications, have been shown to effectively reduce anxiety and depression in patients with PCOS^(21)^. Patients with severe psychological disorders are referred to a mental health specialist for diagnosis and treatment. Integrating mental health care with routine PCOS management can help reduce disease burden and improve overall prognosis.

Multidisciplinary collaborations are growing rapidly in several areas, including diabetes and mental health treatment, to optimize benefits for patients and save resources^(22)^. According to the European Society for the Study of Obesity^(23)^, a multidisciplinary obesity management team should include endocrinologists, nutritionists, exercise specialists, and mental health practitioners. The Chinese Consensus recommends that a team of specialists, including doctors, nurses, dietitians, and exercise experts, provide effective interventions for patients with PCOS to manage infertility and preserve fertility^(24)^.

All obese PCOS patients in this study aimed to reduce their weight through a multidisciplinary approach encompassing lifestyle changes, psychological support, medication, and traditional Chinese medicine. Confidence and persistence are crucial factors for weight loss success. However, long-term strict calorie restrictions can decrease compliance and increase dropout rates, leading to problems such as binge eating and vomiting^(25)^. Additionally, overexercise and improper exercise techniques can cause injury and reduce exercise adherence^(26,27)^. Therefore, a gradual weight reduction is recommended. Each patient should have a personalized, science-based, and sustainable weight loss plan that meets their unique needs and is within a reasonable and scientific scope. Patients struggling with chronic obesity and lacking weight-loss confidence require extra support to feel valued and build self-assurance^(28)^. Case managers should closely monitor progress, provide encouragement, and adjust programs according to each patient’s situation.

At the time of the interview, the patients had been diagnosed with PCOS for 3 months to 7 years; however, only one patient had adequate knowledge of their disease, while the others had minimal understanding. Studies suggest that professional health guidance improves patient adherence and treatment outcomes, as sufficient knowledge and correct awareness help patients better understand their condition, adhere to treatment, and manage their disease more effectively^(29, 30)^. Medical institutions should promote PCOS knowledge by providing scientific, accurate, and authoritative information, both online and offline in simple language. Additionally, society should raise awareness through community health education bulletin boards and regular health education activities. Boosting public health awareness will contribute to a healthier society.

Family support involves providing materials and emotional assistance to patients’ family members^(31)^. In this study, not all patients with obesity, PCOS, and reproductive needs conceived successfully, and most lacked sufficient emotional support from their families, leading to anxiety and depression. Due to infertility challenges, they spent a significant amount of time in clinics^(32)^. Compared to healthy women, patients with PCOS suffer from more negative life events, greater psychological pressure, and less family attention to their psychological needs^(33)^. Harmonious family relationships can effectively help patients buffer their stress and pressure, but they also need to pay attention to the emotional needs of patients’ families and ensure a good atmosphere among them. Hospitals and communities can actively conduct group psychological interventions for patients with PCOS and their families^(34)^. This will encourage family members to pay attention to patients’ mental health status and provide timely emotional support.

## LIMITATIONS AND FUTURE RESEARCH DIRECTIONS

This study has some limitations. First, while it offered valuable insight into the weight management experiences and needs of patients of reproductive age with obesity and PCOS in China, these findings are confined to a specific geographic and cultural context. Thus, these results may not be generalizable to other regions or cultures. Second, the small sample size and reliance on self-reported data may have introduced bias. Future research should involve larger and more diverse samples from various regions to improve the generalizability of our findings.

## CONCLUSION

Weight management is often inadequate among patients with obese PCOS, as they tend to rely on single approaches based on misconceptions. These patients require specialized weight management, including professional guidance and long-term monitoring by case managers. Additionally, cooperation between society and medical institutions is crucial to address both the physical and mental health needs of patients and their families. Policymakers should prioritize early screening and diagnosis of PCOS, integrate metabolic health management, and increase research funding. Public awareness campaigns should promote early diagnosis, raise awareness of symptoms, and encourage weight management. These actions will help address the multifaceted challenges faced by women with PCOS.
