# Supplementary material for: Weight management experiences and needs in obese polycystic ovary syndrome patients of childbearing age in China: a qualitative study
Source: Rev Esc Enferm USP. 2025 Apr 14;59:e20240354. doi: 10.1590/1980-220X-REEUSP-2024-0354en (PMC11999536; doi:10.1590/1980-220X-REEUSP-2024-0354en)
Supplement: Translation

# Translation: Experiencias de control de peso y necesidades de pacientes obesos y de edad reproductiva con síndrome de ovario poliquístico en China: un estudio cualitativo

**DOI:** 10.1590/1980-220X-REEUSP-2024-0354es

**Authors:** Hong Jiang, Min Luo, Hongjin Wu, Xixi Li

**Affiliations:** 1 Chengdu Medical College, School of Nursing, Chengdu, Sichuan, China.; 2 University of Electronic Science and Technology of China, School of Medicine, Nursing Department, Mianyang Central Hospital, Mianyang, Sichuan, China.; 3 903 Hospital, Chengdu Medical College, Jiangyou, Mianyang, Sichuan, China.

**Keywords:** Síndrome del Ovario Poliquístico, Obesidad

## Abstract

**Objetivo:**

Explorar las experiencias de control de peso y necesidades de pacientes obesos y de edad reproductiva con síndrome de ovario poliquístico (SOP) en China.

**Métodos:**

Trece pacientes fueron reclutados en un hospital terciario de la provincia de Sichuan. Entrevistas estructuradas cara a cara fueron realizadas usando un método fenomenológico, con resultados analizados siguiendo los siete pasos de la metodología Colaizzi.

**Resultados:**

Los métodos claves identificados en el control de peso incluyeron la medicación, la dieta, el ejercicio, y la corrección del hábito. Las actitudes de afrontamiento fueron variadas, con algunos pacientes exhibiendo respuestas positivas o negativas. Las experiencias revelaron adherencia a corto plazo al control de la dieta pero dificultad al mantenimiento de rutinas de ejercicio. Los pacientes expresaron la necesidad de apoyo por parte del personal médico y la familia, así como un interés en medicamentos y en medicina tradicional China para ayudar a perder peso.

**Conclusión:**

Las perspectivas en las experiencias del manejo de peso y las necesidades de pacientes obesos con SOP pueden guiar al personal médico en abordar estos desafíos. La atención al bienestar emocional de los pacientes y las familias durante el manejo del peso es crucial para un intervención efectiva.

## INTRODUCCIÓN

La Organización Mundial de la Salud (OMS) define a las mujeres en edad reproductiva como aquellas de entre 15 y 49 años^(1)^. Este grupo demográfico es crítico, ya que su impacto en la salud se extiende más allá de su bienestar individual al de sus familias y comunidades. La OMS hace hincapié en que abordar la salud reproductiva de la mujer es esencial para mejorar los resultados de salud maternoinfantil y alcanzar objetivos sociales y económicos más amplios^(2)^. El Programa para el Desarrollo de la Mujer China (2021–2030)^(3)^ da prioridad a «la mujer y la salud» para mejorar la salud reproductiva y abordar los desafíos a los que se enfrentan las mujeres en edad reproductiva. El objetivo principal de este programa es abordar cuestiones de salud críticas como la infertilidad, un problema crítico con una prevalencia del 5,8% (2006–2010) al 8,1% (2017–2019), que afecta aproximadamente al 10%–15% de las parejas^(4)^. El síndrome de ovario poliquístico (SOP) es la principal causa de infertilidad anovulatoria en hasta el 15 % de las mujeres en todo el mundo y afecta a mujeres en edad reproductiva con una prevalencia global del 6–20%^(5)^. El SOP se caracteriza por irregularidades menstruales, hiperandrogenismo (que se manifiesta como hirsutismo y acné) y ovarios poliquísticos en las ecografías^(6)^. Además de los problemas de salud reproductiva, la resistencia a la insulina relacionada con el SOP aumenta el riesgo de diabetes tipo 2 (hasta un 28 % a los 40 años), así como de hipertensión, colesterol alto y aterosclerosis, todo lo cual eleva el riesgo de enfermedades cardíacas y accidentes cerebrovasculares^(7)^. Estas complicaciones no solo reducen la calidad de vida, sino que también suponen una carga para los sistemas de salud, ya que los cuidados a largo plazo y la medicación aumentan los costes^(8)^. Las pacientes con SOP tienen predisposición a la obesidad, lo que puede agravar su enfermedad^(9)^. La interacción entre el SOP y la obesidad a menudo crea un círculo vicioso, lo que complica aún más los resultados de salud de las personas afectadas y los síntomas. Sin embargo, se ha demostrado que la pérdida de entre un 5 y un 10 % del peso corporal mejora los síntomas del SOP^(10)^. El control eficaz del peso es esencial en pacientes con obesidad y SOP. Aunque existen numerosas estrategias de control del peso para estos pacientes, su adherencia es generalmente baja^(11)^.

Por lo tanto, para comprender mejor las experiencias y las necesidades de control de peso de las mujeres obesas con SOP, este estudio emplea métodos cualitativos, entrevistas en profundidad con este grupo de pacientes. Los conocimientos recopilados proporcionarán recomendaciones para estrategias de atención centradas en el paciente, garantizando que las voces de las mujeres que viven con SOP sean fundamentales para mejorar los resultados de la atención de salud e informar las políticas futuras.

## MÉTODO

### Diseño Y Lugar

Este estudio utilizó un método cualitativo de entrevistas semiestructuradas para conocer las experiencias y necesidades prácticas de las pacientes chinas obesas con SOP en edad reproductiva. Se utilizó el método de análisis de contenido inductivo cualitativo para describir las experiencias y necesidades de control de peso en pacientes obesas con síndrome de ovario poliquístico en edad reproductiva en China. Los resultados se presentaron de acuerdo con los criterios consolidados para la presentación de informes de investigación cualitativa (CO-REQ).

### Métodos

Este estudio empleó un diseño cualitativo para alinearse con su objetivo de explorar las experiencias de las pacientes. Se realizaron entrevistas semiestructuradas con mujeres diagnosticadas con SOP, centrándose en sus desafíos, necesidades y estrategias relacionadas con el control de peso.

### Población, Criterios de Inclusión y Exclusión

Utilizando el método de muestreo intencional, se seleccionaron pacientes obesas con SOP que estaban inscritas en la clínica ambulatoria de ginecología y endocrinología de un hospital general terciario de la provincia de Sichuan en China, entre marzo de 2023 y abril de 2023. Las pacientes eran elegibles para su inclusión en función de los siguientes criterios: 1) pacientes en edad reproductiva diagnosticadas con SOP^(12)^; 2) índice de masa corporal (IMC) ≥30 kg/m^2(13)^; 3) conciencia mental normal y buena capacidad de comunicación. Se excluyó a las pacientes que: 1) se retiraron automáticamente durante la entrevista; 2) no pudieron proporcionar datos de contacto; 3) habían estado embarazadas.

### Entrevista y Procedimiento

A través de la revisión de la literatura, el equipo de investigación diseñó inicialmente el esquema de la entrevista, realizó entrevistas previas con tres pacientes que cumplían los criterios, revisó el esquema de la entrevista de acuerdo con la entrevista y, finalmente, determinó el esquema formal de la entrevista. Las preguntas modificadas de la guía de la entrevista para cada participante se enumeraron en la Tabla 1.

**Tabla 1:** Guía de la entrevista - Mianyang, Sichuang, China, 2023.

Número	Preguntas
1	¿Conoce algo sobre el SOP?
2	¿Actualmente maneja su peso? Si es así, ¿cómo controla su peso?
3	¿Tiene alguna sensación o sentimiento al manejar su peso?
4	¿Cuál es su motivación para la gestión del peso?
5	¿Encuentra alguna dificultad en su proceso de gestión del peso?
6	¿Cuál es la efectividad de su programa actual de control de peso?
7	¿Hay algo en lo que necesite ayuda con respecto a la gestión del peso?

Este estudio empleó entrevistas semiestructuradas individuales utilizando un enfoque fenomenológico. Antes de las entrevistas, el investigador comunicó el propósito y la metodología del estudio a los encuestados, quienes luego dieron su consentimiento informado. Todas las entrevistas se realizaron en una sala de archivo para pacientes ambulatorios tranquila y privada y duraron aproximadamente 30 minutos. Antes de la entrevista, se aseguró a los entrevistados que su participación no afectaría la calidad de su tratamiento futuro. Se utilizó una grabadora de voz para grabar las entrevistas, y un investigador capacitado siguió una guía de entrevista preestablecida. El investigador se mantuvo neutral, se abstuvo de hacer preguntas capciosas o evaluaciones sesgadas, y evitó interrumpir a los participantes. Durante la entrevista, el investigador prestó atención a las expresiones faciales y los movimientos corporales de los sujetos durante la entrevista, y grabó oportunamente.

### Análisis de Datos

Después de cada entrevista, dos profesionales capacitados trabajaron de forma independiente para transcribir las grabaciones de audio en material textual en un plazo de 24 horas. Se utilizó el software NVIVO 12 para organizar y gestionar los datos textuales. El análisis siguió el método de análisis de siete pasos de Colaizzi^(14)^ para procesar los datos de forma sistemática. El primer autor revisó los datos originales de la entrevista para identificar temas preliminares. Estos temas se compararon luego con los derivados de forma independiente por los dos profesionales capacitados. Las discrepancias se discutieron y resolvieron de forma colaborativa, lo que dio lugar a una serie de temas propuestos. Por último, todos los miembros del equipo de investigación revisaron los temas propuestos y llegaron a un consenso para establecer los temas finales.

### Aprobación Ética

Todos los métodos se llevaron a cabo de acuerdo con las directrices y normativas pertinentes o de acuerdo con la Declaración de Helsinki. La aprobación del Comité de Ética del Hospital Central de Mianyang, Facultad de Medicina, Universidad de Ciencia y Tecnología Electrónica de China antes de realizar el estudio (ID: S20230306-02). Este estudio fue aprobado por el Comité de Ética e Investigación Clínica del Hospital, con exención concedida sobre la necesidad de consentimiento informado.

## RESULTADOS

### Características Demográficas de los Participantes

El tamaño de la muestra de este estudio se basó en la saturación de información, y finalmente se incluyó un total de 13 pacientes obesas con SOP. En este estudio, de acuerdo con el principio de confidencialidad, se utilizaron D1–D13 para representar a los entrevistados, y la información general de los entrevistados se muestra en la Tabla 2.

**Tabla 2:** Información general sobre los entrevistados (n = 13) - Mianyang, Sichuang, China, 2023.

Número	Edad (años)	Nivel educativo	Estado civil y reproductivo	Ocupación	Necesidad reproductiva actual
D1	30	Graduada de escuela secundaria superior	Casada con hijos	Desempleada	No
D2	35	Graduada de escuela secundaria superior	Casada con hijos	Empleada en una organización no gubernamental	Sí
D3	24	Graduada de escuela secundaria inferior	Casada sin hijos	Empleada en una organización no gubernamental	Sí
D4	18	Estudiante de escuela secundaria superior	Soltera y sin hijos	Estudiante	No
D5	20	Estudiante universitario	Soltera y sin hijos	Estudiante	No
D6	26	Graduada de escuela secundaria superior	Casada sin hijos	Empleada en una organización no gubernamental	Sí
D7	25	Graduada universitario	Casada sin hijos	Desempleada	Sí
D8	28	Graduada universitario	Casada con hijos	Comerciante	No
D9	20	Estudiante universitario	Soltera y sin hijos	Estudiante	No
D10	27	Graduada de escuela secundaria superior	Soltera y sin hijos	Comerciante	Sí
D11	23	Graduada universitario	Soltera y sin hijos	Empleada gubernamental	No
D12	20	Estudiante universitario	Soltera y sin hijos	Estudiante	No
D13	23	Candidata a maestría	Soltera y sin hijos	Estudiante	No

Mediante un análisis detallado de las entrevistas transcritas, se identificaron cuatro temas: los métodos de control de peso en pacientes obesas con SOP, las actitudes de las pacientes obesas con SOP ante el control de peso, las experiencias de las pacientes obesas con SOP con las prácticas de control de peso y las necesidades de control de peso de las pacientes obesas con SOP, como se muestra en la Tabla 3.

**Tabla 3:** Temas clave y subtemas - Mianyang, Sichuang, China, 2023.

Temas	Subtemas
Métodos de gestión del peso en pacientes con SOP obeso	1. Manejo de la medicación 2. Manejo de la dieta 3. Manejo del ejercicio 4. Corrección de hábitos irrazonables
Actitud de afrontamiento hacia la gestión del peso en pacientes con SOP obeso	1. Actitud de afrontamiento positiva 2. Actitud de afrontamiento negativa
Experiencias con los métodos de gestión del peso en pacientes con SOP obeso	1. Fácil adherirse al control dietético a corto plazo 2. Difícil adherirse al ejercicio
Necesidades de gestión del peso en pacientes con SOP obeso	1. Necesitan ayuda del personal médico 2. Necesitan ayuda de sus familias 3. Desean usar medicamentos para ayudar en la reducción de peso 4. Desean usar la medicina tradicional china para ayudar en la reducción de peso

### Tema 1: Los Métodos de Control de Peso en Pacientes Obesas con Sop

#### Subtema 1: Control de la Medicación

Algunos entrevistados tenían una combinación de resistencia a la insulina o diabetes mellitus tipo 2, y sus médicos les recetaron medicamentos como metformina y simeticona para controlar la afección, lo que también ayudó a controlar el peso. La mayoría de los pacientes informaron de efectos positivos en el control del peso, aunque un pequeño grupo mostró un escaso cumplimiento de la medicación prescrita. Además, algunos pacientes habían comprado medicamentos para adelgazar a través de canales no oficiales, pero este grupo experimentó un aumento de peso tras dejar de utilizarlos.

*Fui a propósito a un* médico de medicina china, que me *recetó algunos medicamentos chinos orales y tópicos. (D2)*

*Antes de pesar solo 72 kg, me sentía muy gorda y compré medicamentos para adelgazar (en Internet) para comer, como resultado, ahora he aumentado a 85 kg. (D10)*

*El médico me recetó metformina e inyecciones de semaglutida, dijo que estos medicamentos me ayudan a controlar el azúcar en sangre y a perder peso, hasta ahora es muy eficaz, he perdido 7,5 kg. (D11)*

*Mi médico me recetó metformina, pero a menudo me olvido de tomar la medicación. (D13)*

#### Subtema 2: Control de la Dieta

Todas las entrevistadas de este estudio realizaron cambios en su dieta (incluidos ajustes en la estructura de los alimentos, restricciones calóricas y cambios en los horarios de las comidas, etc.) después de que se les diagnosticara SOP. Sin embargo, seguían careciendo de conocimientos profesionales sobre nutrición.

*Solía ser muy aficionada a alimentos como el arroz y los fideos. Desde que me diagnosticaron SOP, ahora como un tazón pequeño de arroz porque ellos (amigos cercanos) dijeron que no es bueno consumir demasiados alimentos con almidón. (D1) Ahora, solo bebo leche de soja y como huevos y pan integral por la mañana, pechuga de pollo y un poco de arroz y muchas verduras para el almuerzo, y básicamente pepino, berenjena, tomates y lechuga para la cena. (D2)*

*Después de que el* médico me *diera el alta, como más ligero. (D4)*

*En comparación con antes, ahora como menos calorías en total, pero todavía no estoy segura de cómo hacerlo exactamente. (D7)*

#### Subtema 3: Gestión del Ejercicio

Solo una de las entrevistadas en este estudio se marcó un programa de ejercicio y lo siguió regularmente a diario. Por el contrario, el resto de las entrevistadas hacían poco o ningún ejercicio.

*No hago ejercicio de forma deliberada porque estoy ocupada la mayor parte del tiempo. (D1)*

*Hago ejercicio todos los días. Me impongo la regla de caminar* más de 6000 *pasos y saltar a la cuerda* más de 100 *veces al día. (D2)*

*De vez en cuando hago ejercicio, pero en general no le dedico mucho tiempo. (D9)*

*No solía hacer ejercicio, pero después de descubrir esta enfermedad (síndrome de ovario poliquístico), empecé a hacer gimnasia, pero solo cuando no estoy en el trabajo, así que solo puedo hacer gimnasia unas 3 o 4 veces al mes durante media hora cada vez. (D11)*

#### Subtema 4: Corrección de Hábitos Irracionales

La mayoría de los encuestados en este estudio creían subjetivamente que habían mejorado hábitos problemáticos, como beber más agua, picar menos, limitar el alcohol y dormir más temprano.

Sin embargo, aunque muchos reconocieron y mejoraron algunos de sus hábitos poco saludables, no los corrigieron todos

*Ahora como muy pocas patatas fritas y tomo muy poco té con leche, de todos modos, como algo de vez en cuando. (D5)*

*Estoy en el negocio y a menudo como en restaurantes. Pero ahora bebo menos porque tengo que hablar de negocios con otros y es imposible no beber. (D10)*

*Ya no fumo, a veces todavía bebo algo de cerveza, solía acostarme después de las 3 de la mañana, pero ahora me acuesto antes de la 1. (D13)*

### Tema 2: Actitud de Afrontamiento del Control de Peso en Pacientes Obesas con Sop

#### Subtema 1: Actitud Positiva Para Afrontar la Situación

Muchos de los encuestados indicaron que el control del peso es beneficioso para la recuperación de la enfermedad y destacaron la importancia de cooperar con sus médicos. También creían que mantener una mentalidad positiva es crucial para el éxito de la pérdida de peso a largo plazo. Además, ver la mejora en el aspecto de otros pacientes tras perder peso con éxito les motivó a continuar con sus esfuerzos. *Controlar el peso es difícil. De todos modos, es una batalla larga, pero tengo mucha confianza. Esto se debe a que la gente todavía necesita ejemplos positivos, y quiero ser un ejemplo para mis hijos. (D1)*

*Lo* más importante para *mí es mi salud, y entiendo lo que tengo que hacer para mantener mi peso bajo control. Si lo controlo bien, no tendré diabetes, cáncer de endometrio y todo eso. (D2) Solo quiero estar delgada y lucir hermosa. Antes había una paciente en* línea que *también tenía esta enfermedad. Ella perdió peso y se volvió muy atractiva. (D4)*

#### Subtema 2: Actitud Negativa Para Hacer Frente a la Situación

Todos los encuestados en este estudio eran pacientes con obesidad crónica. Algunos habían perdido la confianza en su capacidad para perder peso debido a su larga lucha contra la obesidad y a los fracasos anteriores, y ya no creían que fuera posible para ellos perder peso con éxito. Por lo tanto, estos pacientes eran reacios a participar en el control del peso.

*No importa lo que haga, no puedo perder peso desde hace muchos años. (D10)*

*De hecho, siento que como más o menos lo mismo que la gente de mi entorno con peso normal, pero estoy* más gorda que *los demás, así que me parece injusto. Así que no creo que haya ninguna necesidad de perder peso, y ya está. (D11)*

*Soy una persona gorda muy común, he sido gorda desde que era niña, solo me gusta comer y dormir, no quiero hacer ejercicio, de todos modos no importa, no me voy a casar ni a tener hijos. (D12)*

### Tema 3: Experiencias con Métodos de Control de Peso en Pacientes Obesas con Sop

#### Subtema 1: Fácil Adherencia al Control Dietético a Corto Plazo

La mayoría de las pacientes dijeron que estaban muy dispuestas a perder peso mediante el control de la dieta y que podían seguir dietas estrictas a corto plazo. Pero les gustaría seguir un plan dietético a largo plazo, y que la supervisión de otra persona sería mejor para ayudarles a seguir su dieta.

*Me gustaría poder picar algo un par de veces al mes si es posible, de lo contrario siempre me entrarán antojos. (D4)*

*Ahora no como nada por la noche. (D10)*

*Solía tomar té con leche y picar, pero ahora ya no. Llevo dos meses sin hacerlo, pero no puedo seguir así a largo plazo sin picar. (D11)*

*En el colegio me vigilan mis amigos, en casa mi madre. (D12)*

#### Subtema 2: Dificultad Para Seguir el Ejercicio

La mayoría de los pacientes tienen dificultades para seguir el ejercicio. Los oficinistas a menudo evitan el ejercicio debido a la tensión física y mental de sus trabajos, mientras que los estudiantes luchan por encontrar tiempo debido a las exigencias académicas. Algunos pacientes interrumpen el ejercicio por razones físicas, psicológicas o urgentes.

*Estoy demasiado cansada después del trabajo, así que no quiero hacer ejercicio. (D3)*

*Ahora estoy en mi tercer año de instituto, así que tengo que pasar mucho tiempo estudiando, es imposible que haga ejercicio todos los días. (D4)*

*Hay muchas cosas en la vida real que pueden alterar tus planes, como que de repente haya algo en casa o algo así, y eso hace que deje de hacer ejercicio. (D8)*

*Me lesioné la rodilla haciendo ejercicio antes y ahora no me atrevo a hacer ejercicio sola. (D9)*

### Tema 4: Necesidades de Control de Peso en Pacientes Obesas Con Sop

#### Subtema 1: Necesidad de Ayuda por Parte del Personal Médico

Todas las pacientes expresaron su deseo de recibir apoyo por parte del personal médico, de obtener más conocimientos sobre la enfermedad, de conocer el impacto de la obesidad en la enfermedad y de aumentar la confianza en la reducción de peso y el éxito.

*Me han diagnosticado SOP desde hace 7 años. Para ser sincera, puedo perder peso muy* rápido, pero *luego recuperarlo* más *adelante. Sigo pensando que no es bueno para mi salud, debería tener que venir a ver al médico al hospital con regularidad. (D2)*

*No sé específicamente cuáles son las consecuencias de mi enfermedad, solo sé que si no puedo perder peso ahora no podré quedarme embarazada fácilmente. Me gustaría perder peso rápidamente, así que me gustaría hacer* más preguntas a *los* médicos y *enfermeras del hospital sobre lo que debería hacer ahora mismo. (D3)*

*De todos modos, he probado todas las formas que conozco para perder peso por mi cuenta, y ahora realmente no puedo hacer nada más, por eso he venido al hospital para perder peso. (D12)*

#### Subtema 2: Necesidad de Ayuda Para sus Familias

El apoyo familiar inadecuado para algunos pacientes conduce a impedimentos para perder peso y emociones anormales debido a la falta de atención de la familia.

*Cuando estaba perdiendo peso, mi familia me atraía a propósito con alguna comid a deliciosa.*

*Me gustaría que los miembros de mi familia no hicieran eso cuando estoy perdiendo peso. (D5)*

*Siento que a los miembros de mi familia solo les importa cuándo puedo quedar embarazada, y les he dicho que necesito reducir mi peso antes de poder tener un bebé más fácilmente. Sin embargo, solo me presionan para que quede embarazada más rápido. (D7)*

*Cuido de mi hijo todos los días, no tengo tiempo para hacer ejercicio , antes quería ir a un campamento cerrado para perder peso, pero cuando fui allí nadie cuidó de mi hijo. (D8)*

#### Subtema 3: Deseo Utilizar Medicamentos Para Ayudar A Reducir el Peso

Algunos pacientes expresaron su deseo de que se les recetaran medicamentos para perder peso. Querían perder peso controlando su apetito y aumentando su metabolismo.

*Solo necesito que me receten pastillas para adelgazar ahora, no puedo esperar a perder peso, quiero quedarme embarazada pronto. (D6)*

*Quiero perder peso rápidamente ahora, es mejor que me receten pastillas para adelgazar, nunca puedo perder peso por mi cuenta. (D10)*

*Espero que me recete algún medicamento que pueda controlar mi apetito. Antes seguía una dieta muy estricta, pero a veces como demasiado y luego lo lamento, y voy a vomitar. (D11)*

#### Subtema 4: Deseo Utilizar la Medicina Tradicional China Para Ayudar a Reducir El Peso

La mayoría de los pacientes expresaron su deseo de recibir ayuda de los profesionales de la medicina tradicional china, ya que creen que tratamientos como la acupuntura, la terapia de puntos de acupuntura y los alambres enterrados podrían ayudar a perder peso. Además, pensaban que remojar los pies en medicamentos chinos a base de hierbas especialmente formulados podría ayudar a estimular el metabolismo.

*Mucha gente dice que la medicina tradicional china puede regular la menstruación y ayudar a perder peso. (D1)*

*La medicina tradicional china es realmente útil. Tengo una amiga que también tiene SOP, le han recetado algunas medicinas chinas de un* médico chino, *ahora no solo tiene la menstruación normal, sino que también se ha vuelto* más *joven y más guapa. Y sobre todo, ha bajado de peso. (D2)*

*Hace poco me hice una sesión de acupuntura en un centro de medicina tradicional china, me dijeron que la acupuntura puede ayudarme a perder peso, y de momento sí que he perdido peso. (D12)*

## DISCUSIÓN

Los estudios han revelado que las mujeres con SOP experimentan diversos grados de ansiedad (37 %) y depresión (42 %), y factores como la obesidad y los intentos fallidos previos de perder peso aumentan significativamente su carga psicológica ^(15, 16)^. Todas las entrevistadas en este estudio eran pacientes obesas con SOP, que habían intentado previamente programas de pérdida de peso sin éxito y habían recuperado peso. La ansiedad y la depresión entre las pacientes con SOP a menudo se manifiestan de diversas maneras, incluyendo sentimientos persistentes de tristeza, desesperanza e inutilidad. Estas pacientes también pueden experimentar síntomas como trastornos del sueño, dificultad para concentrarse y preocupación excesiva por su salud y su futuro. Otro estudio destacó que la angustia psicológica grave se asociaba con un mayor riesgo de autolesión y una menor calidad de vida^(17)^. Estos problemas de salud mental también pueden exacerbar la disfunción endocrina, reducir las tasas de concepción y afectar negativamente a la calidad de vida en general^(18)^. Por lo tanto, es fundamental evaluar e intervenir sistemáticamente lo antes posible cuando los pacientes presentan síntomas de ansiedad y depresión para reducir la carga de la enfermedad y mejorar el pronóstico.Sin embargo, los estudios han revelado que solo un pequeño porcentaje de médicos en China abordan activamente los problemas psicológicos de los pacientes con SOP^(19)^. Esta brecha puede atribuirse a su gran base de población, al número significativo de pacientes y a la insuficiencia de recursos médicos, lo que hace que los médicos presten atención solo a las enfermedades físicas de los pacientes y descuiden sus condiciones psicológicas. Es imposible realizar entrevistas en profundidad a todos los pacientes con obesidad y SOP para determinar su estado psicológico. Se recomienda que todas estas pacientes se sometan a un examen psicológico de rutina utilizando herramientas estandarizadas como la Escala de Trastorno de Ansiedad Generalizada o el Cuestionario de Salud del Paciente^(20)^. Se deben realizar exámenes repetidos basados en el juicio clínico y la progresión de la enfermedad, particularmente en momentos críticos (por ejemplo, cuando las pacientes muestran cambios de humor o alcanzan hitos significativos del tratamiento). Las intervenciones oportunas y específicas son esenciales para las pacientes con estados emocionales anormales. Se ha demostrado que los enfoques terapéuticos basados en la evidencia, incluida la terapia cognitivo-conductual y las intervenciones en el estilo de vida, como el ejercicio estructurado y las modificaciones dietéticas, reducen eficazmente la ansiedad y la depresión en pacientes con SOP^(21)^. Las pacientes con trastornos psicológicos graves son derivadas a un especialista en salud mental para su diagnóstico y tratamiento. La integración de la atención de la salud mental en el tratamiento rutinario del SOP puede ayudar a reducir la carga de la enfermedad y mejorar el pronóstico general.

Las colaboraciones multidisciplinares están creciendo rápidamente en varias áreas, incluyendo el tratamiento de la diabetes y la salud mental, para optimizar los beneficios para los pacientes y ahorrar recursos^(22)^. Según la Sociedad Europea para el Estudio de la Obesidad^(23)^, un equipo multidisciplinar de gestión de la obesidad debe incluir endocrinólogos, nutricionistas, especialistas en ejercicio y profesionales de la salud mental. El Consenso chino recomienda que un equipo de especialistas, incluidos médicos, enfermeros, dietistas y expertos en ejercicio, ofrezca intervenciones eficaces para que los pacientes con SOP controlen la infertilidad y preserven la fertilidad^(24)^.

Todas las pacientes obesas con SOP de este estudio tenían como objetivo reducir su peso mediante un enfoque multidisciplinar que abarcaba cambios en el estilo de vida, apoyo psicológico, medicación y medicina tradicional china. La confianza y la persistencia son factores cruciales para el éxito en la pérdida de peso. Sin embargo, las restricciones calóricas estrictas a largo plazo pueden disminuir el cumplimiento y aumentar las tasas de abandono, lo que conduce a problemas como atracones y vómitos^(25)^. Además, el exceso de ejercicio y las técnicas de ejercicio inadecuadas pueden causar lesiones y reducir la adherencia al ejercicio^(26, 27)^. Por lo tanto, se recomienda una reducción de peso gradual. Cada paciente debe tener un plan de pérdida de peso personalizado, basado en la ciencia y sostenible que satisfaga sus necesidades únicas y esté dentro de un alcance razonable y científico. Las pacientes que luchan contra la obesidad crónica y carecen de confianza en sí mismas para perder peso requieren un apoyo adicional para sentirse valoradas y desarrollar seguridad en sí mismas^(28)^. Los gestores de casos deben monitorear de cerca el progreso, brindar aliento y ajustar los programas de acuerdo con la situación de cada paciente.

En el momento de la entrevista, a las pacientes se les había diagnosticado SOP entre 3 meses y 7 años antes; sin embargo, solo una paciente tenía un conocimiento adecuado de su enfermedad, mientras que las demás tenían una comprensión mínima. Los estudios sugieren que la orientación de salud profesional mejora la adherencia de los pacientes y los resultados del tratamiento, ya que un conocimiento suficiente y una concienciación correcta ayudan a los pacientes a comprender mejor su afección, a adherirse al tratamiento y a controlar su enfermedad de forma más eficaz^(29, 30)^. Las instituciones médicas deben promover el conocimiento del SOP proporcionando información científica, precisa y autorizada, tanto en línea como fuera de línea, en un lenguaje sencillo. Además, la sociedad debe concienciar a través de tablones de anuncios de educación de salud comunitaria y actividades periódicas de educación de salud. El aumento de la concienciación sobre la salud pública contribuirá a una sociedad más sana.El apoyo familiar implica proporcionar materiales y asistencia emocional a los familiares de los pacientes^(31)^. En este estudio, no todos los pacientes con obesidad, SOP y necesidades reproductivas concibieron con éxito, y la mayoría carecía de suficiente apoyo emocional de sus familias, lo que les provocaba ansiedad y depresión. Debido a los problemas de infertilidad, pasaban una cantidad significativa de tiempo en clínicas^(32)^. En comparación con las mujeres sanas, las pacientes con SOP sufren más acontecimientos negativos en la vida, mayor presión psicológica y menos atención familiar a sus necesidades psicológicas^(33)^. Las relaciones familiares armoniosas pueden ayudar eficazmente a las pacientes a amortiguar el estrés y la presión, pero también es necesario prestar atención a las necesidades emocionales de las familias de las pacientes y garantizar un buen ambiente entre ellas. Los hospitales y las comunidades pueden llevar a cabo activamente intervenciones psicológicas grupales para las pacientes con SOP y sus familias^(34)^. Esto animará a los miembros de la familia a prestar atención al estado de salud mental de los pacientes y a proporcionarles apoyo emocional de manera oportuna.

## LIMITACIONES E INVESTIGACIONES FUTURAS

Este estudio tiene algunas limitaciones. En primer lugar, aunque ofreció una valiosa información sobre las experiencias y necesidades de control de peso de las pacientes en edad reproductiva con obesidad y SOP en China, estos hallazgos se limitan a un contexto geográfico y cultural específico. Por lo tanto, estos resultados pueden no ser generalizables a otras regiones o culturas. En segundo lugar, el pequeño tamaño de la muestra y la dependencia de los datos autoinformados pueden haber introducido sesgos. Las investigaciones futuras deberían incluir muestras más grandes y diversas de varias regiones para mejorar la generalizabilidad de nuestros hallazgos.

## CONCLUSIÓN

El control del peso suele ser inadecuado entre las pacientes con SOP obesas, ya que tienden a depender de enfoques únicos basados en conceptos erróneos. Estas pacientes requieren un control de peso especializado, que incluya orientación profesional y un monitoreo a largo plazo por parte de los administradores de casos. Además, la cooperación entre la sociedad y las instituciones médicas es crucial para atender las necesidades de salud física y mental de las pacientes y sus familias. Los responsables políticos deberían dar prioridad a la detección y el diagnóstico tempranos del SOP, integrar la gestión de la salud metabólica y aumentar la financiación de la investigación. Las campañas de concienciación pública deberían promover el diagnóstico precoz, concienciar sobre los síntomas y fomentar el control del peso. Estas acciones ayudarán a abordar los múltiples desafíos a los que se enfrentan las mujeres con SOP.
